# Automated vs manual delineations of regions of interest- a comparison in commercially available perfusion MRI software

**DOI:** 10.1186/1471-2342-12-16

**Published:** 2012-07-18

**Authors:** Ivana Galinovic, Ann-Christin Ostwaldt, Carina Soemmer, Helena Bros, Benjamin Hotter, Peter Brunecker, Jochen B Fiebach

**Affiliations:** 1 Center for Stroke Research Berlin, Charité-Universitätsmedizin Berlin, Campus Benjamin Franklin, Hindenburgdamm 30, 12200, Berlin, Germany; 2 International Graduate Program Medical Neurosciences, Charite-Universitätsmedizin, Berlin, Luisenstrasse 56, 10117, Berlin, Germany; 3 Charité-Universitätsmedizin, Berlin, Charitéplatz 1, 10117, Berlin, Germany

**Keywords:** Magnetic resonance imaging, Perfusion MRI, Acute ischemic stroke

## Abstract

**Background:**

In perfusion magnetic resonance imaging a manual approach to delineation of regions of interest is, due to rater bias and time intensive operator input, clinically less favorable than an automated approach would be. The goal of our study was to compare the performances of these approaches.

**Methods:**

Using Stroketool, PMA and Perfscape/Neuroscape perfusion maps of cerebral blood flow, mean transit time and Tmax were created for 145 patients with acute ischemic stroke. Volumes of hypoperfused tissue were calculated using both a manual and an automated protocol, and the results compared between methods.

**Results:**

The median difference between the automatically and manually derived volumes was up to 210 ml in Perfscape/Neuroscape, 123 ml in PMA and 135 ml in Stroketool. Correlation coefficients between perfusion volumes and radiological and clinical outcome were much lower for the automatic volumes than for the manually derived ones.

**Conclusions:**

The agreement of the two methods was very poor, with the automated use producing falsely exaggerated volumes of hypoperfused tissue. Software improvements are necessary to enable highly automated protocols to credibly assess perfusion deficits.

## Background

The increased use of the perfusion imaging (PI) – diffusion-weighted imaging (DWI) mismatch hypothesis [1] in studies of acute ischemic stroke (AIS) and clinical practice [1,2] is raising demands from software packages developed for volumetric calculations of hypoperfusion. Typically these programs offer the possibility to delineate a region of interest (ROI) manually but also, to a varying extent, automatically. Manual approaches to delineation are biased and require time intensive operator input. Therefore a mostly automated procedure, if accurate, would be preferred in clinical practice. The goal of our study was comparing the automated approach to the manual approach while using a number of different software packages.

## Methods

The study design and cohort characteristics have been previously reported [3]. Briefly, using Stroketool (Digital Image Solutions, Germany, http://www.digitalimagesolutions.de), PMA (v3.2.0.4, ASIST, Japan, http://asist.umin.jp/index-e.htm) and Perfscape/Neuroscape (Olea Medical SAS, France, http://www.olea-medical.com) perfusion maps of cerebral blood flow (CBF), mean transit time (MTT) and Tmax were calculated for 145 patients imaged within 24 hours of AIS. The inclusion criteria were: clinically and radiologically confirmed AIS, hypoperfusion on the initial PI examination (assessed by the attending neuroradiologist) and availability of a follow-up MRI scan. For each parameter map, three thresholds were applied. The Tmax thresholds were 4, 6 and 8 seconds of delay [4] and the MTT thresholds were 5, 6 and 8 seconds. As no uniform CBF scale was available, the three CBF thresholds were different across software. Both MTT and CBF thresholds were chosen empirically using a random sample of acute stroke patients. ROI volumes were calculated using a manual and an automated protocol. In the automated protocol, once the thresholds have been applied, no further post-processing was done. Maps created in PMA and Stroketool also underwent a second post-processing step in SPM8 (Wellcome Trust Centre for Neuroimaging, UK) to cut away scalp and spaces filled with cerebrospinal fluid (CSF). This was not necessary for Perfscape/Neuroscape due to the program’s implemented filtering. In the manual protocol a human rater excluded, from the thresholded maps, areas unlikely to reflect credible hypoperfusion.

Radiological outcome was defined as the final lesion volume on follow-up FLAIR images. Clinical outcome was defined as the National Institute of Health Stroke Scale (NIHSS) score at the time of hospital discharge. All statistics were done in PASW Statistics 18. Analyses of correlations were performed using the Spearman signed-ranks correlation test.

## Results

**Figure 1:**
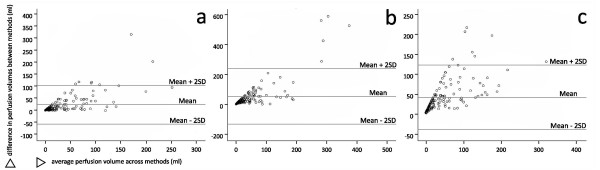
**Bland-Altman plots for the top performing automatic map and threshold, without additional CSF filtering for PMA and Stroketool a- Perfscape/Neuroscape; b- PMA and c- Stroketool.** The mean and the limits of agreement (mean +/−2 standard deviations) are shown as solid lines. The difference in perfusion volumes was calculated as the manually derived volume subtracted from the automated one

The median final lesion volume was 6.55 ml (IQR 0.8 – 31.6 ml). The median difference between the automatically and manually derived volumes was up to 210 ml (between 90% and 386%) in Perfscape/Neuroscape, 123 ml (between 192% and 1415%) in PMA and 135 ml (between 357% and 815%) in Stroketool. (Table 1). Bland-Altman plots for agreement of methods are shown in Figure 1. All correlation coefficients between volumes of perfusion deficit and radiological and clinical outcome were considerably lower for the automatic volumes than for the manually derived ones. The top performing automated map was Tmax in all three programs. With additional filtering applied to automated volumes calculated in PMA and Stroketool the median difference between the automatically and manually derived volumes dropped down to a maximum of 64.91 ml (between 44% and 238%) and 67.28 ml (between 33% and 369%), respectively (Table 1).

**Table 1:** Difference of ROI volumes between the automated and the manual protocol

**Program**	**Perfscape/Neuroscape**	**PMA**	PMA with filtering *	**Stroketool**	Stroketool with filtering *
	**Median**	**Median**	**Median**	**Median**	**Median**
	**IQR**	**IQR**	**IQR**	**IQR**	**IQR**
Parameter					
Tmax 4 s	25.80	123.68	64.91	122.89	37.50
	12.8–66.1	75.2–206.8	27.3–138.1	75.6–176.5	14.9–75.3
Tmax 6 s	12.74	29.48	4.83	53.18	7.75
	4.1–34.4	16.4–67.8	1.0–22.4	34.0–82.6	1.8–18.3
Tmax 8 s	8.05	23.16	2.68	31.78	1.67
	2.6 - 28.1	13.5 – 55.4	0.4 – 15.8	16.1 – 50.1	0.2 – 6.5
MTT 5 s	186.78	35.12	11.59	129.35	58.74
	151.4–248.5	17.8–87.8	3.4–44.1	82.5–225.7	21.8–130.5
MTT 6 s	154.40 114.8–207.0	16.76 8.4–49.0	4.34 0.9–18.4	80.18 49.1–143.6	26.51 8.0–63.7
MTT 8 s	94.24	7.36	0.77	33.82	6.07
	55.0–143.3	3.8–17.4	0.3–3.9	20.9–57.6	1.6–18.8
CBF highest threshold	209.92	54.35	23.05	134.50	67.28
	160.0–262.8	27.5–107.9	7.6–64.4	89.9–193.0	39.0–110.2
CBF medium threshold	151.93	36.14	10.51	87.28	35.27
	97.6–212.2	18.6–65.9	3.7–36.5	59.4–134.0	18.1–61.7
CBF lowest threshold	63.66	23.40	4.33	53.95	12.73
	39.6–119.4	13.3–39.3	1.4–15.3	37.2–83.0	6.6–28.4

CBF indicates cerebral blood flow; MTT mean transit time; IQR interquartile range. All values are in ml. * Values indicate volumes after additional CSF filtering in SPM8.

## Discussion

The median volume of the perfusion deficit varied greatly based on the map and threshold and even across software for the same map and threshold. This discrepancy could in part be explained through the different choice of AIFs, the use of different deconvolution techniques, different implementations of the same calculation algorithm as well as differences in motion correction across different programs [5-7]. Our group had already conducted a study on a group of patients with no ischemia, using the same three software packages, and observed the presence of numerous artifacts; typically the cortex proximal to the skull and infratentorial cerebral and cerebellar tissue [8]. Programs without implemented CSF filtering also suffered from artifacts such as ventricles, eyeballs and scalp [8]. As expected, the same artifacts were present in our current patient cohort. This explains the weaker correlation coefficients and overshoot of the automated delineations as compared to the manual ones. Although most of the values on the Bland-Altman difference plot fall within the limits of agreement (Figure 1), these are much too broad with regards to the cohort’s median lesion volumes and the fact that, based on location, even a lesion of a few ml can be clinically significant. Additional CSF filtering greatly reduced the differences between the automatic and the manual volumes, pointing to a need for implemented filtering.

## Conclusion

In conclusion, current automated use of the here evaluated programs would lead to falsely exaggerated volumes of hypoperfused tissue in patients with AIS. However a number of improvements, such as algorithms for judging perfusion asymmetry between hemispheres and allowing selection of the vessel territory of expected hypoperfusion, could aid automated protocols in credibly assessing perfusion deficits.

## Competing interests

The authors have no conflicts of interest to declare. All vendors provided software free of charge, no author received grants or fees from any of the involved software right owners and companies.

## Authors’ contributions

IG gathered the patient cohort, partly analyzed the imaging data, performed statistical analysis and drafted the manuscript. ACO participated in the analysis of the imaging data. CS participated in the analysis of the imaging data. HB participated in the analysis of the imaging data. BH participated in the gathering of the patient cohort and the drafting of the manuscript. PB performed the automated segment of data analysis. JBF was responsible for supervision and co-drafted the final version of the manuscript with IG. All authors read and approved the final manuscript.

## Pre-publication history

The pre-publication history for this paper can be accessed here:

http://www.biomedcentral.com/1471-2342/12/16/prepub
